# Longitudinal association between dietary protein intake and survival in peritoneal dialysis patients

**DOI:** 10.1080/0886022X.2023.2182605

**Published:** 2023-03-02

**Authors:** Shu-Hong Bi, Xiaoxiao Wang, Wen Tang, Tao Wang, Baohua Li, Chunyan Su

**Affiliations:** a Department of Nephrology, Peking University Third Hospital, Beijing, China; b Research Center of Clinical Epidemiology, Peking University Third Hospital, Beijing, China; c Nursing Department, Peking University Third Hospital, Beijing, China

**Keywords:** Malnutrition, daily protein intake, peritoneal dialysis, survival, nitrogen balance

## Abstract

**Background:**

Decreased dietary protein intake (DPI) may lead to protein-energy malnutrition and may be associated with increased mortality risk. We hypothesized that longitudinal changes in dietary protein intake have independent associations with survival in peritoneal dialysis (PD) patients.

**Methods:**

668 stable PD patients were selected in the study from January 2006 to January 2018 and were followed up until December 2019. Their three-day dietary records were collected at the baseline (the sixth month after PD) and thereafter every 3 months for two and a half years. The latent class mixed models (LCMM) were used to identify subgroups of PD patients with similar longitudinal trajectories of DPI. The relation between DPI (baseline and longitudinal data) and survival was examined using Cox model to estimate death hazard ratios. Meanwhile, different formulae were used to assess nitrogen balance.

**Results:**

The results showed that baseline DPI ≤ 0.60g/kg/day was associated with the worst outcome in PD patients. Patients with DPI 0.80–0.99g/kg/day and DPI ≥ 1.0g/kg/day both presented positive nitrogen balance; patients with DPI 0.61–0.79g/kg/day presented obviously negative nitrogen balance. Longitudinal association between time-dependent DPI and survival was found in PD patients. The consistently low DPI' (0.61–0.79g/kg/d) group was correlated with increased death risk as compared with the 'consistently median DPI' group (0.80–0.99g/kg/d, HR = 1.59, *p* = 0.008), whereas there was no difference in survival between 'consistently median DPI' group and 'high-level DPI' group (≥1.0 g/kg/d, *p* > 0.05).

**Conclusion:**

Our study revealed that DPI ≥ 0.8 g/kg/day was beneficial to the long-term outcome for the PD population.

## Introduction

Protein-energy malnutrition is a serious problem related to poor outcomes in peritoneal dialysis (PD) patients [1–3]. Previous studies have shown that the prevalence of malnutrition in continuous ambulatory peritoneal dialysis (CAPD) patients is 18–56% [4–6]. Decreased dietary protein intake (DPI) may lead to protein-energy malnutrition and may be associated with poor survival [7].

An appropriate recommendation of DPI in PD patients is of great importance. In the National Kidney Foundation Kidney Disease Outcomes Quality Initiative (K/DOQI) clinical practice guidelines for nutrition in chronic kidney disease in 2020, guideline 3 states that, ‘In adults with chronic kidney disease (CKD) 5D on maintenance hemodialysis (1 C) or PD (OPINION) who are metabolically stable, we recommend prescribing a dietary protein intake of 1.0–1.2 g/kg body weight per day to maintain a stable nutritional status’ [8]. However, the level of evidence is not high, further studies and more evidence are needed in this area.

Moreover, this recommendation for DPI is greater than the actual protein intake of the majority of PD patients as indicated by almost all dietary protein surveys [6,9,10]. Many published studies also suggested that patients with PD could maintain good nutritional status with a protein intake of 0.8 g/kg/d [11–16]. Meanwhile, many prior studies that examined the association between DPI and survival evaluated only the effect of the initial or baseline DPI, obtained at the start of the study, on subsequent mortality [15,17,18]. There is still a paucity of controlled metabolic studies, as well as long-term well-designed clinical trials, studying protein intake in PD patients.

We, therefore, conducted this study to explore the appropriate DPI level with regard to outcome events using nitrogen balance and examine whether there were longitudinal associations between time-varying daily protein intake and survival in a group of chronic PD patients.

## Methods

### Subjects and follow-up

It was a retrospective cohort study of kidney failure patients who underwent CAPD therapy at Peking University Third Hospital from January 2006 to January 2018 and were followed up until December 2019.

Stable CAPD patients were selected in this study. Eligible patients were those who 1) had been on PD for at least 6 months; 2) were over 18 years old; 3) visited PD clinic and got dietary intakes evaluated regularly. Patients with congestive heart failure (New York Heart Association functional class II to IV), malignancy, or pregnancy, were excluded. Demographic data below were collected: gender, age, etiology of ESRD, body weight, height, presence of diabetes mellitus (DM), and Charlson index [19]. All patients were followed until death, transfer to hemodialysis (HD), kidney transplantation, or the end of the study. We recorded all-cause mortality as outcome events.

The study was approved by the Medical Ethical Committee of Peking University Third Hospital. Informed consent was waived because of the retrospective design of this study.

### Dietary variables

We recommend prescribing a dietary protein intake of at least 0.8 g/kg of protein and 25 kcal/kg of energy based on previous clinical findings in the Chinese PD population and the guideline from EBPG [13,15,16,20]. For patients with decreased dietary intake, the doctors would check whether they had some complications and gave them treatment accordingly. The dietitian would provide nutritional counseling, encourage them to increase their dietary intake, and prescribe some nutrients as needed.

During the follow-up, all patients were asked to visit PD clinic regularly with their three-day dietary records. Three-day dietary records at the sixth month of dialysis were collected as baseline data and were collected every three months for two and a half years. After checking using food models by the dietitian, the information of the food diary was inputted into a computer software program (PD Information Management System, Peking University Third Hospital, and then the dietary intake was calculated accordingly. Nitrogen intake (NI) was calculated as protein intake (g/d)/6.25. DPI and daily energy intake (DEI) were normalized by ideal body weight (IBW), which was defined as body height (cm) minus 105 (modified Broca method) [21].

### Dialysis adequacy and nitrogen balance at baseline

We asked our patients to record the glucose concentration and volume of instilled dialysate 1 day before the clinic visit. Meanwhile, their 24-h urine and dialysate samples were collected to measure urea and creatinine levels. Using standard methods, we further calculated weekly total Kt/V urea, creatinine clearance, and residual kidney function (RKF). It has been demonstrated that there is a linear relationship between the urea nitrogen appearance (UNA, the urea nitrogen output in urine and dialysate) and the total nitrogen appearance (TNA, the nitrogen output in urine, feces, and dialysate) [22,23]. TNA was calculated using the Bergstrom formula [24] or new formulae which were shown as: TNA = 1.5 + 1.24 UNA [25]. NB (nitrogen balance) =NI -TNA.

### Blood chemistries

Baseline biochemical parameters, such as blood urea nitrogen, creatinine, hemoglobin, albumin, phosphate, potassium, and sodium were measured by routine laboratory procedures.

### Statistical analyses

Continuous variables were reported as either mean ± standard deviation (SD) or median (interquartile range, IQR) based on their distribution. For the statistical analysis, we used one-way ANOVA to compare the results of nitrogen balance with different baseline DPI levels. Categorical data were presented as proportions.

Patient follow-up time was computed as the time of starting PD to the date of outcome events including all-cause mortality or censored at transplantation, transfer to HD or other centers, or the end of the study, whichever was earliest. Survival time was defined as the time from the patient’s start of PD to the occurrence of endpoint events or to the end of follow-up.

The latent class mixed models (LCMM) were fitted using the R ‘lcmm’ package [26], which offered a way to identify subgroups of PD patients with similar DPI trajectories. Patients who survived at least two and a half years with at least one record during follow-up were included in this analysis. Models of 1 to 4 latent classes were repeatedly fitted with the number of latent classes in a stepwise fashion. Best fit was assessed *via* model fit statistics (Akaike information criterion, AIC; Bayesian information criterion, BIC; sample size adjusted BIC, SABIC) and clinical judgment. Each participant was assigned to the latent class with the highest membership probability. Logistic regression models were used to determine the influencing factors according to latent classes. One-way ANOVA and chi-square tests were used to explore the association between baseline characteristics and longitudinal DPI trajectories. Logistic regression models were then used to determine the influencing factors of longitudinal DPI trajectory latent classes.

The Cox proportional hazards model of univariate and multivariate analysis was used to evaluate the association between baseline DPI, longitudinal DPI trajectories subgroups, and survival as well as to estimate the Hazard ratios (HR) with 95% Confidence Interval (CI). Potential confounders included patients’ baseline demographic data, data of dietary intake, dialysis adequacy, residual kidney function, laboratory tests and nitrogen balance.

The Kaplan-Meier analysis was used to generate survival curves. The log-rank test was used to evaluate the difference in survival rate according to baseline DPI and longitudinal DPI groups.

All of the reported *p*-values were two-tailed, and statistical significance was set at 0.05. Statistical analyses were performed using the SPSS software package (SPSS version 21.0, Chicago, IL, USA) and R software, version 4.1.2 (R Foundation for Statistical Computing).

## Results

### Baseline characteristics of the study population

Totally 986 patients who started CAPD therapy in our center from January 2006 to January 2018 were initially followed up. Within half a year of dialysis, 124 dialysis patients withdrew from PD. Three patients had complete recovery of kidney function, 191 patients could not be followed-up regularly due to living far away from PD center, suffering from cancer, et al. Finally, 668 patients were included in this study, 343 males and 325 females, mean age 58.88 ± 16.03 years. 452 patients survived at least 2 years and had at least one DPI record during follow-up. According to the etiology of PD patients, the percentage of diabetic kidney disease (DKD), chronic glomerulonephritis (CGN), hypertensive nephropathy (HTN), chronic interstitial nephritis (CIN) and polycystic kidney was 33.8%, 31.3%, 15.1%, 8.9%, and 3.1%, respectively. 254 patients (38.0%) had diabetes.

The median follow-up duration was 6.9 years in the overall cohort. The 1-year, 2-year, 3-year, 5-year survival rates were 96.7%, 87.6%, 78.3%, and 60.3%, respectively. At the end of the study, 184 patients were still on PD, 299 died, 72 had transferred to HD, 29 had undergone kidney transplantation, 84 had transferred to other hospitals, and the causes of death were cardiovascular diseases (113 cases, 37.8%), systemic infections (77 cases, 25.8%), multiple organ failure (38 cases, 12.7%), malignant tumors (18 cases, 6.0%) and other causes (53 cases, 17.7%).

The dietary intake, nutritional status, and nitrogen balance of the patients were shown in Table 1. Their mean DPI was 0.86 ± 0.25g/kg/day; their mean DEI was 25.45 ± 6.86 kcal/kg/day.

**Table 1.** Baseline DPI, nutritional parameters and dialysis adequacy of 668 PD patients.

Variables	Data
height, cm	163.60 ± 8.49
body weight, kg	63.42 ± 12.55
BMI	23.58 ± 3.71
Charlson index	6 (4,7)
DPI, g/kg/day	0.86 ± 0.25
DEI, kcal/kg/day	25.45 ± 6.86
Fat intake, g/d	53.23 (43.25, 65.79)
Carbohydrate intake, g/d	195.80 (155.90, 237.76)
Alb, g/l	37.54 ± 4.68
Dialysis dose, ml	6762.38 ± 1765.05
ultrafiltration, ml	360.00 (132.50,650.00)
urine volume, ml	550.00 (250.00,950.00)
total fluid removal, ml	994.50 (700.00,1317.50)
Kt/V	1.99 ± 0.62
Tccr (L/w)	59.16 (47.15, 75.23)
eGFR, ml/min	2.40 (1.11, 3.97)
Nitrogen intake, g/d	8.02 ± 2.48
TNA, g/d (Bergstrom)	9.55 ± 2.08
TNA, g/d (New formula)	8.01 ± 2.14
NB, g/d (Bergstrom)	−1.52 ± 2.65
NB, g/d (New formula)	0.01 ± 2.68
Hb, g/l	115.90 ± 19.43
Serum, creatinine, umol/l	718.07 ± 256.71
Serum urea, mmol/l	20.62 ± 6.00
Serum calcium, mmol/l	2.25 ± 0.29
Serum phosphate, mmol/l	1.52 ± 0.41
Serum potassium, mmol/l	4.30 ± 0.75
Serum sodium, mmol/l	139.20 ± 3.13
CO_2_CP, mmol/l	26.93 ± 3.71

DPI: dietary protein intake; PD: peritoneal dialysis; BMI: body mass index; DEI: dietary energy intake; Alb: albumin; Tccr: total clearance rate of creatinine; eGFR: estimated Glomerular filtration rate; TNA: total nitrogen appearance; NB: nitrogen balance; Hb: hemoglobin; CO_2_CP: carbon dioxide combining power.

### Results of nitrogen balance and the effect of baseline DPI level on survival

#### Baseline DPI level as a continuous variable

In the univariate Cox regression model, a higher level of DEI was significantly associated with a lower risk of death (HR 0.963; 95% CI 0.946 to 0.981, *p* < 0.001). Based on the analysis of the univariate Cox regression model and multivariate Cox proportional hazard analysis, old age, higher Charlson index, hypoalbuminemia, and a lower level of blood sodium were significantly associated with inferior survival. Baseline DPI and nitrogen balance were not related to PD patients’ survival (Table 2).

**Table 2.** Univariate and multivariate Cox regression analysis of the impact of baseline data on mortality.

Variables	Hazard ratios	95% CI	*p* value
univariate Cox regression analysis			
Age, years	1.055	1.045–1.066	<0.001
gender, male vs. female	0.965	0.769–1.211	0.761
Charlson index, points	1.419	1.340–1.504	<0.001
DM, yes vs. no	1.796	1.429–2.258	<0.001
DPI, g/kg/day	0.634	0.391–1.029	0.065
DEI, kcal/kg/day	0.963	0.946–0.981	<0.001
Serum creatinine, umol/l	0.998	0.998–0.999	<0.001
Serum urea, mmol/l	0.961	0.941–0.982	<0.001
Serum calcium, mmol/l	0.950	0.646–1.399	0.797
Serum phosphate, mmol/l	0.909	0.678–1.219	0.523
Serum sodium, mmol/l	0.934	0.901–0.969	<0.001
Serum potassium, mmol/l	0.684	0.579–0.809	<0.001
CO_2_CP, mmol/l	0.994	0.962–1.026	0.711
Alb, g/l	0.923	0.903–0.944	<0.001
Hb, g/l	0.998	0.992–1.004	0.528
N balance, g/d (New formula)	1.032	0.989–1.078	0.149
eGFR, ml/min	1.003	0.956–1.052	0.917
Kt/V	1.029	0.847–1.250	0.775
Multivariate Cox regression analysis^a^			
Age, years	1.032	1.017–1.046	<0.001
Charlson index, points	1.161	1.059–1.272	0.002
DEI, kcal/kg/day	0.981	0.963–1.001	0.057
Serum sodium, mmol/l	0.960	0.926–0.994	0.022
Serum potassium, mmol/l	0.862	0.730–1.018	0.081
Alb, g/l	0.959	0.933–0.986	0.003

DM: diabetes; DPI: dietary protein intake; DEI: dietary energy intake; CO_2_CP: carbon dioxide combining power; Alb: albumin; Hb: hemoglobin; N: nitrogen; eGFR: estimated Glomerular filtration rate.

^a^Adjusted for DM, DPI, Serum creatinine, Serum urea.

#### Baseline DPI level as a categorical variable

**Figure 1.**
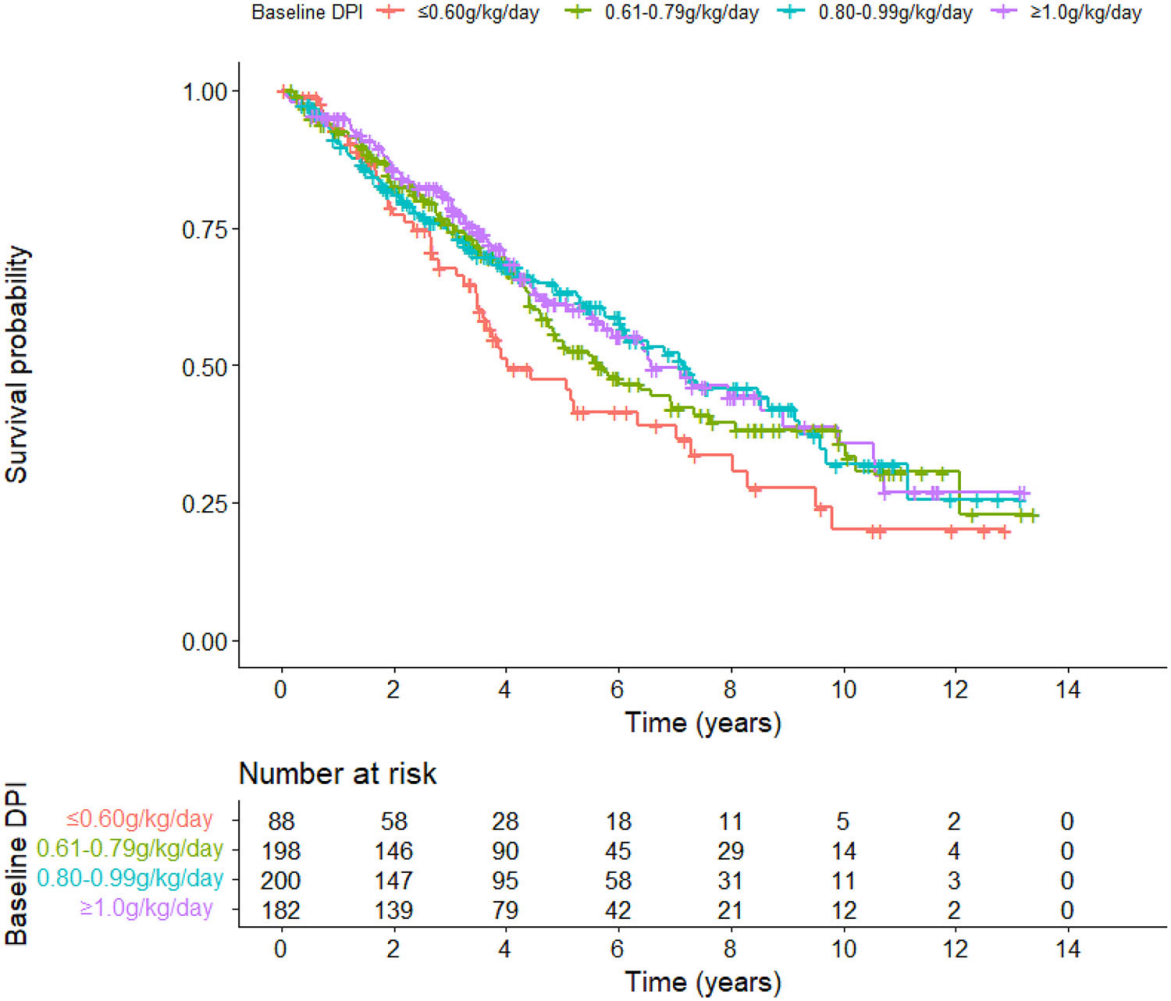
The Kaplan–Meier survival curves in PD patients according to the baseline DPI level. PD: peritoneal dialysis; DPI: dietary protein intake.

All the subjects were classified into four groups according to baseline DPI, i.e., ≤0.60 g/kg/day, 0.61–0.79 g/kg/day, 0.80–0.99 g/kg/day, and ≥1.0 g/kg/day. As shown in Figure 1, Kaplan–Meier analysis was used to analyze the survival. The results showed inferior survival in patients with the lowest DPI group (DPI ≤ 0.60g/kg/day) compared to the other three groups (*p* < 0.05). Meanwhile, there was no significant difference in survival among the three groups (DPI 0.61–0.79 g/kg/day, 0.80–0.99 g/kg/day, and ≥1.0 g/kg/day, *p* > 0.05).

Results of nitrogen balance showed that there was a significant difference among the four groups and between any two groups (*p* < 0.001). When using the Bergstrom formula, only patients with DPI ≥ 1.0g/kg/day presented positive nitrogen balance (0.64 ± 2.62g/d) and patients in the other three groups presented negative nitrogen balance (-4.04 ± 1.80 g/d, -2.59 ± 1.97 g/d, and -1.10 ± 1.95 g/d, respectively). Results from the new formula showed that patients with DPI 0.80-0.99g/kg/day and DPI ≥ 1.0g/kg/day both presented positive nitrogen balance (0.44 ± 2.00 g/d and 2.15 ± 2.66 g/d, respectively), and patients with DPI 0.61–0.79g/kg/day and DPI ≤ 0.60g/kg/day both presented obviously negative nitrogen balance (-1.05 ± 2.02 g/d and -2.48 ± 1.85 g/d, respectively).

### The effect of longitudinal DPI trajectories on survival

#### Longitudinal DPI trajectories subgroups

**Figure 2.**
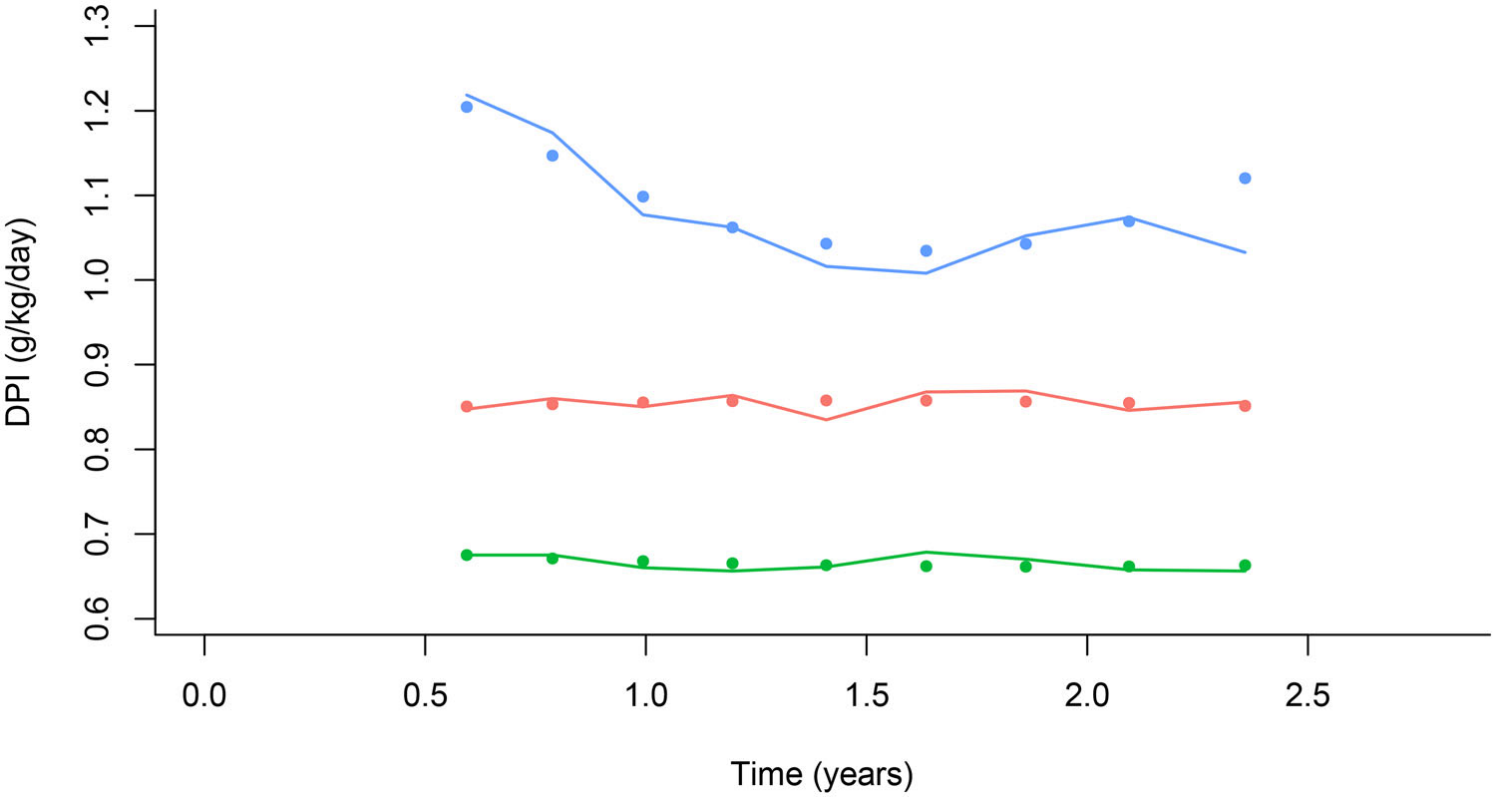
Grouping of time-dependent DPI in PD patients. Red, the consistently median DPI group (0.80–0.99 g/kg/day, *n* = 253); Green, the consistently low DPI group (0.61–0.79 g/kg/day, *n* = 154); Blue, the high DPI group (≥1.0 g/kg/day, *n* = 45). PD: peritoneal dialysis; DPI: dietary protein intake.

Among 668 patients, 452 patients who survived at least 2 years and had at least one DPI record during follow-up, were included in the analysis. LCMM models with up to four latent classes were fitted (see Supplemental Table 1 for the goodness of fit indices and Supplemental Figure 1), and the 3-class model was selected as the optimal solution. Figure 2 showed the class-specific trajectories of DPI levels after PD. Of all the 452 participants, 253 (56.0%) of patients showed a ‘consistently median DPI’ trajectory (class 1, 0.80–0.99 g/kg/day), 154 (34.1%) fell into the ‘consistently low’ DPI score category (class 2, 0.61–0.79 g/kg/day). Class 3 included 45 (9.9%) patients, and the value of DPI showed a decreasing tendency till the 12^th^ month and then kept stable and high levels (≥1.0 g/kg/day).

The associations between baseline characteristics and belonging to each latent class for DPI levels are shown in Table 3 and Table 4. Patients with higher Charlson index and negative nitrogen balance were more likely to belong to the ‘consistently low DPI’ class than in the ‘consistently median DPI’ class. Patients with DPI ≥ 1.0 g/kg/day had higher Kt/v levels, serum sodium levels, and more positive nitrogen balance.

**Table 3.** Baseline characteristics of PD patients according to longitudinal DPI latent classes.

Variables	0.80-0.99g/kg/day *n* = 253	0.60-0.79 g/kg/day *n* = 154	≥1.0 g/kg/day *n* = 45	statistic	*p*
Age, years	57.6 ± 15.3	58.7 ± 14.8	55.5 ± 16.8	0.796	0.452
BMI, kg/m^2^	23.8 ± 3.8	23.7 ± 3.6	23.1 ± 3.4	0.687	0.504
Charlson index, points	5.3 ± 1.9	5.8 ± 2.2	5 ± 2.3	4.846	0.008
DM, *n* (%)	79 (31.3%)	71 (46.1%)	12 (26.7%)	10.900	0.004
Hb, g/l	116.5 ± 19.3	118.2 ± 18	117.2 ± 15.1	0.408	0.665
Low Alb, *n* (%)	59 (23.3)	29 (18.8)	11 (24.4)	1.317	0.518
eGFR, ml/min	2.8 ± 2.2	2.9 ± 2.5	4 ± 2.9	4.976	0.007
Tccr, L/w	63.5 ± 20.9	66.1 ± 51.8	72.7 ± 27.7	1.365	0.257
Kt/v	2 ± 0.5	1.9 ± 0.6	2.3 ± 0.7	6.594	0.002
Sodium, mmol/l	139.4 ± 3	139.4 ± 3.2	140.6 ± 2.9	3.353	0.036
Potassium, mmol/l	4.4 ± 0.7	4.4 ± 0.8	4.3 ± 0.7	1.092	0.336
NBs, g/d (New formula)	0.1 ± 2.6	−0.7 ± 2.7	1.1 ± 2.6	10.056	<0.001

PD: peritoneal dialysis; DPI: dietary protein intake; BMI: body mass index; DM: diabetes; Hb: hemoglobin; low Alb: low albumin (<35 g/l); eGFR: estimated glomerular filtration rate; Tccr: total clearance rate of creatinine; NBs: nitrogen balance by new formula; CO_2_CP: carbon dioxide combining power.

**Table 4.** Associations between baseline characteristics and being in latent classes for DPI levels using logistic regression model.

Variables	0.60–0.79 g/kg/day *n* = 154		≥1.0 g/kg/day *n* = 45	
	OR (95%CI)	*p*	OR (95%CI)	*p*
DM	1.488 (0.930,2.380)	0.097	1.054 (0.461,2.410)	0.902
eGFR, ml/min	1.003 (0.895,1.125)	0.957	1.101 (0.934,1.298)	0.251
Kt/v	0.680 (0.418,1.106)	0.120	2.047 (1.006,4.162)	0.048
Na, mmol/l	1.014 (0.947,1.087)	0.687	1.155 (1.027,1.299)	0.016
NBs, g/d	0.857 (0.786,0.933)	<0.001	1.193 (1.055,1.350)	0.005
Charlson index, points	1.142 (1.018,1.281)	0.023	0.893 (0.746,1.068)	0.215

DPI: dietary protein intake; DM: diabetes; Hb: hemoglobin; Alb: Albumin; eGFR: estimated Glomerular filtration rate; Na: serum sodium; K: serum potassium; NBs: nitrogen balance by new formula.

Reference group: 0.80–0.99g/kg/day (*n* = 253).

#### Longitudinal DPI trajectories and survival

**Figure 3.**
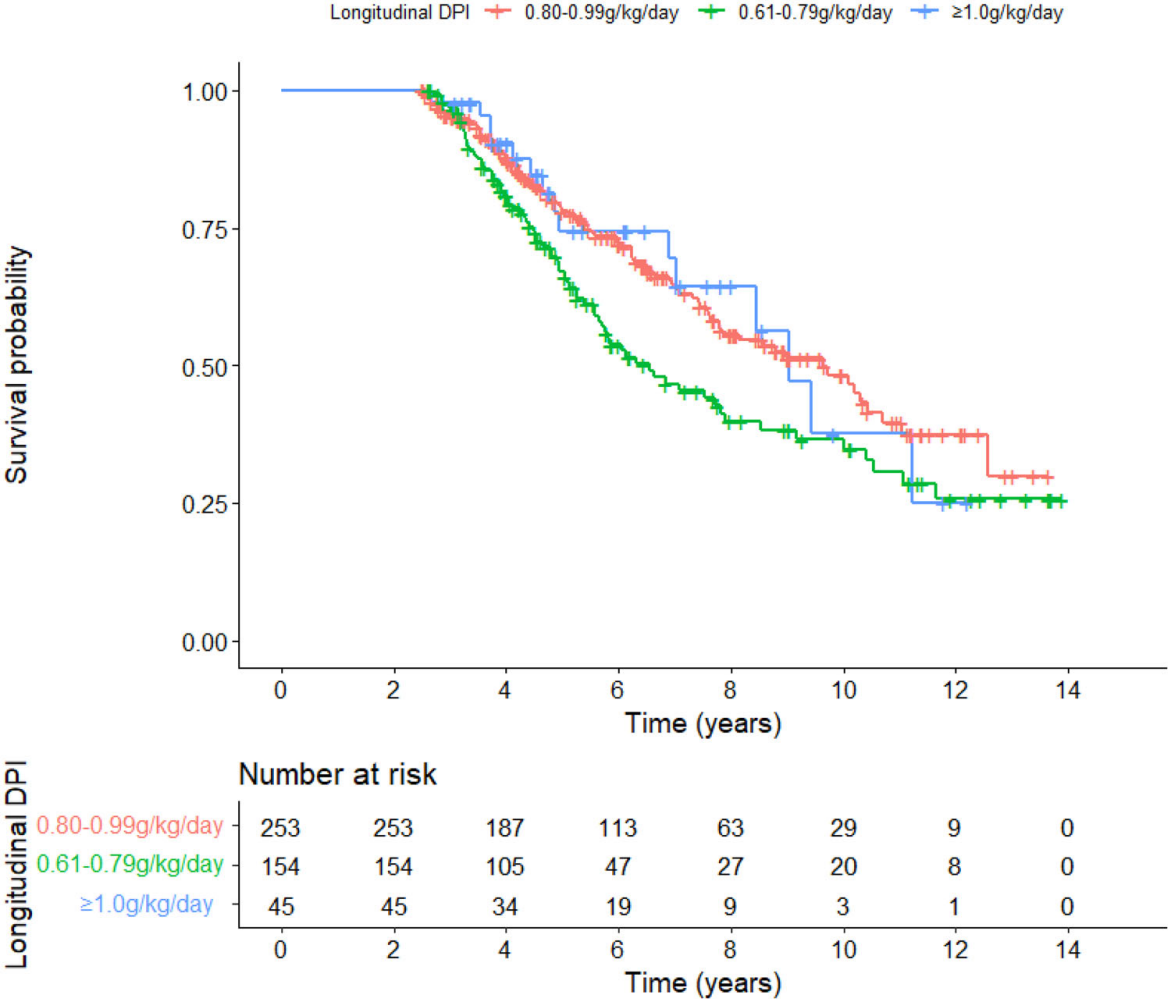
The Kaplan-Meier survival curves in PD patients according to the grouping of time-dependent DPI. PD, peritoneal dialysis; DPI, dietary protein intake.

During follow-up, 181 of the 452 patients died, including 92 of 253 patients in group 1 (0.80-0.99 g/kg/day), 74 of 154 patients in group 2 (0.61–0.79 g/kg/day), and 15 of 45 patients in group 3 (≥1.0 g/kg/day). Kaplan-Meier survival analysis showed that there was a significant difference in survival among the three groups (*p* = 0.027, as shown in Figure 3). The risk of mortality was significantly higher in group 2 compared with group 1 (HR = 1.497, *p* = 0.010), and group 1 and group 3 had a similar risk of mortality (HR = 0.989, *p* = 0.969). After adjusting for age, DM, Charlson index, baseline albumin, Kt/V, estimated glomerular filtration rate (eGFR), and other factors (serum sodium, potassium, nitrogen balance, and DEI), subgrouping of longitudinal DPI levels were significantly associated with survival. The survival of patients in group 2 (0.61–0.79 g/kg/day) was significantly worse than that in group 1 (0.80–0.99 g/kg/day, HR = 1.59, *p* = 0.008). There was no significant difference in the risk of death between group 1 and group 3 (HR = 1.16, *p* = 0.631).

## Discussion

In this study, we explored appropriate DPI levels in CAPD patients based on the results of nitrogen balance and survival analysis. DPI ≥ 0.8 g/kg/day was supposed to be beneficial to the long-term outcome for the PD population. Meanwhile, we examined the longitudinal association between longitudinal daily protein intake trajectories and survival in PD patients. The ‘consistently low DPI’ group (0.61–0.79 g/kg/day) had worse outcomes in comparison with the ‘consistently median DPI’ group (0.80–0.99 g/kg/day). Patients in the ‘consistently median DPI’ group did not show significant differences in terms of survival compared to the high DPI level group (≥1.0 g/kg/day).

This is a long-term study to explore DPI levels with regard to outcome events in PD patients and examine time-varying DPI and survival in PD patients; PD patients enrolled early from January 2006 were followed up until December, 2019. Our findings recommended appropriate DPI level and reinforced the importance of the change of DPI in PD patients.

The results showed that baseline DPI ≤ 0.60g/kg/day was associated with the worst outcome in PD patients. Although there was no significant difference in survival among the three groups (DPI 0.61–0.79 g/kg/day, 0.80–0.99 g/kg/day, and ≥ 1.0g/kg/day), results of nitrogen balance showed that patients with DPI 0.61–0.79 g/kg/day presented negative nitrogen balance. Theoretically, in the long run, DPI 0.61–0.79 g/kg/day in PD patients may lead to malnutrition. Based on the calculation from the Bergstrom formula [24], only patients with baseline DPI ≥ 1.0g/kg/day presented positive nitrogen balance. When we used new formulae to calculate nitrogen balance [25], patients with DPI 0.80–0.99g/kg/day and DPI ≥1.0 g/kg/day both presented positive nitrogen balance.

Classical method for assessing dietary protein requirements is nitrogen balance [27]. Protein equivalent of total nitrogen appearance (PNA) is a common tool used to estimate protein intake and is calculated using urea clearance from 24-h urine and dialysate collection in PD patients. Therefore, the calculation of nitrogen balance and the formulae which were used were of great importance. Bergstrom formula originated from the data of European PD patients and may have overestimated DPI in Asian CAPD patients [18,28]. Recently, new formulae for calculating PNA were generated based on nitrogen balance studies of stable Chinese CAPD patients [25]. Since the study population had a fairly typical Asian body type and the dialysis regimen was consistent with the characteristics of CAPD clinical practice in Asia, the new formulae might be more suitable for DPI estimation in the study population [25]. Therefore, in this study, we also used new formulae to calculate nitrogen balance. Results from new formulae suggested that patients with DPI 0.80–0.99g/kg/day and DPI ≥ 1.0g/kg/day both presented positive nitrogen balance. Therefore, taking into account these results, DPI ≥ 0.8 g/kg/day was supposed to be more appropriate for the long-term outcome in the Asian CAPD population.

These findings were in agreement with other published studies suggesting that patients with PD can maintain good nutritional status with a protein intake of 0.8 g/kg/d [11,13]. In Chinese CAPD patients, Dong et al. reported that, patients with baseline DPI ≤0.73 g/kg/day had the worst outcome [16], which is also consistent with our results. Moreover, another study suggested that patients were diagnosed as well-nourished using subjective global assessment, their DPI levels were only 0.81 g/kg/day [29]. Additionally, 1-year follow-up study suggested that PD patients with 0.8 g/kg/day were in good condition during follow-up [14]. Clearly, the results in our longitudinal cohort study provided more convincing evidence for the DPI recommendation in the PD population. Furthermore, the baseline time was set as the 6^th^ month after the start of PD, which could exclude many unstable patients and eliminate some confounding factors which may affect survival. In the first 6 months, patients were not always clinically stable and may have been suffering from acute superimposed illnesses [30,31].

Another important result from this study is that the longitudinal association between time-varying daily protein intake and survival was found in PD patients. As mentioned above, the ‘consistently median DPI’ group (0.80–0.99 g/kg/day) had better survival than those with lower DPI (0.61–0.79 g/kg/day), and had a similar risk of mortality in comparison with the high DPI level groups (≥1.0 g/kg/day). The longitudinal results confirmed the better outcome with DPI ≥0.80 g/kg/day in CAPD patients.

Trend of time-varying daily protein intake seemed more important to be predictive of patients’ survival in chronic PD patients. Dialysis patients would possibly suffer from some comorbidities and gastrointestinal symptoms such as nausea, vomiting, and loss of appetite. Therefore, a single assessment of dietary protein intake would be inappropriate and insufficient [32–34]. The dynamic changes and trends in DPI may more accurately reflect patients’ regular dietary intake and their disease status. Subgrouping of longitudinal DPI levels was significantly associated with survival. Patients with consistently low DPI (0.61–0.79 g/kg/day) over time correlated with increased death risk. Meanwhile, behind the low level of DPI, patients possibly suffer from some complications such as malnutrition, fluid overload, inflammation, mental disorders, et al. which may cause gastrointestinal discomfort and a decrease in appetite [32,35–37]. Altered taste and smell, side effects of therapeutic drugs, inability to prepare food and feed oneself, impaired mobility, and bad mood all can contribute to the decrease of DPI [36]. We noticed that in our study, patients in DPI 0.61–0.79 g/kg/day group were more likely to have negative nitrogen balance, and higher Charlson index, which may explain low DPI and inferior survival of these patients. We should pay more attention to the population whose dietary protein intake was consistently low and find out the causes. Prompt detection of complications and provision of correct treatment is of great value to improve the survival of PD patients.

Finally, we noticed that a higher baseline level of DEI was significantly associated with better survival in PD patients, which reminds us that we need to pay attention to dialysis patients’ daily energy intake simultaneously. Energy metabolism may be impaired in patients with CKD. Hence, maintaining adequate energy intake is necessary to prevent protein-energy wasting [8,38]. Recent guidelines and review articles also suggest that energy intake in the range of 25 to 35 kcal/kg per day is appropriate to maintain a neutral nitrogen balance and nutritional status in dialysis patients [36,39]. In the present study, the baseline mean total energy intake would be about 30 Kcal/kg/d if we took into account peritoneal dialysate glucose absorption (about 5 Kcal/kg/d) [40], that means the majority of our PD patients could meet the guideline recommendation in terms of energy intake. It probably was one of the main reasons for good nutritional status and survival in this study population.

There are some limitations in this study. The three-day dietary recall data may underestimate the dietary intake, even though we gave all the patients and their caregivers sufficient education. Moreover, the study was conducted in a single center, which might have some limitations in terms of generalizability. Third, some potential confounding factors which may affect the outcomes could not be completely excluded. Similar to previous studies, all estimates in our study were obtained assuming the missing mechanism was missing at random (MAR). Additionally, excluding patients who survived for less than 2 years might lead to selection bias. However, this analysis provided some clues on how to achieve better outcomes for patients who are expected to survive at least 2 years. Our study had several strengths. The longitudinal study with a large cohort provided more scientific evidence on the DPI recommendation in the PD population. Furthermore, time-varying daily protein intake instead of a single evaluation of DPI at the baseline would be more valuable and convincing in the prediction of patients’ survival. Meanwhile, we set the baseline time as the 6^th^ month after the start of PD and eliminated many unstable patients in the first 6 months of PD, which may minimize the impact of some confounding factors.

## Conclusions

Our study revealed that DPI ≥ 0.8g/kg/day was beneficial to the long-term outcome for PD population based on the results of nitrogen balance and survival analysis. Meanwhile, we found that there was a longitudinal association between time-dependent DPI and survival in PD patients, patients with 'consistently low DPI' (0.61–0.79g/kg/d) had the highest mortality.

## Supplementary Material

Supplemental Material

Supplemental Material

## Data Availability

Data are not available due to the property rights of the healthcare center that has collected these data. Chunyan Su could be contacted if someone wants to request the data.
