# Supplementary material for: Longitudinal association between dietary protein intake and survival in peritoneal dialysis patients
Source: Ren Fail. 2023 Mar 2;45(1):2182605. doi: 10.1080/0886022X.2023.2182605 (PMC9987727; doi:10.1080/0886022X.2023.2182605)
Supplement: Supplemental Material [file IRNF_A_2182605_SM5903.pdf]

Table S1. Model fit statistics for LCMM models considering one to four classes.

Number of Classes	AIC	BIC	SABIC	Number of patients assigned to each class			
				1	2	3	4
1	-443.643	-402.506	-434.242	452			
2	-482.868	-421.163	-468.768	404	48		
3	-503.688	-421.414	-484.887	154	253	45	
4	-501.156	-398.314	-477.655	138	55	223	36

LCMM, latent class mixed models; AIC, Akaike information criterion; BIC, Bayesian information criterion; SABIC, sample size adjusted BIC

Information Classification: General
