# Supplementary figures and images for: Longitudinal association between dietary protein intake and survival in peritoneal dialysis patients
Source: Ren Fail. 2023 Mar 2;45(1):2182605. doi: 10.1080/0886022X.2023.2182605 (PMC9987727; doi:10.1080/0886022X.2023.2182605)

Figure S1. Model fit statistics for LCMM models considering one to four classes.

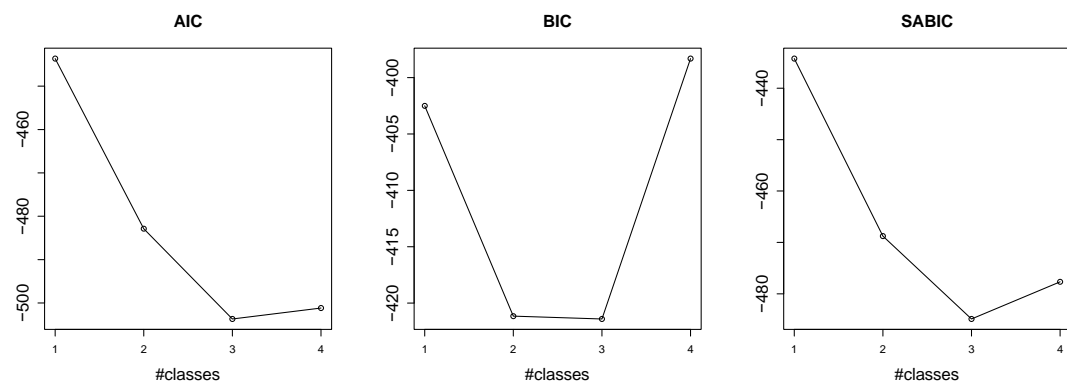

Supplement: Supplemental Material [file IRNF_A_2182605_SM5801.pdf]
